# Relative Localization and Circumnavigation of a UGV0 Based on Mixed Measurements of Multi-UAVs by Employing Intelligent Sensors

**DOI:** 10.3390/s24072347

**Published:** 2024-04-07

**Authors:** Jia Guo, Minggang Gan, Kang Hu

**Affiliations:** State Key Laboratory of Intelligent Control and Decision of Complex Systems, School of Automation, Beijing Institute of Technology, Beijing 100081, China; aganbit@126.com (M.G.); 3120215456@bit.edu.cn (K.H.)

**Keywords:** multi-UAVs, UGV0, RL, UWB sensor, airborne sensor, optical flow sensor, switching topology, circumnavigation

## Abstract

Relative localization (RL) and circumnavigation is a highly challenging problem that is crucial for the safe flight of multi-UAVs (multiple unmanned aerial vehicles). Most methods depend on some external infrastructure for positioning. However, in some complex environments such as forests, it is difficult to set up such infrastructures. In this paper, an approach to infrastructure-free RL estimations of multi-UAVs is investigated for circumnavigating a slowly drifting UGV0 (unmanned ground vehicle 0), where UGV0 serves as the RL and circumnavigation target. Firstly, a discrete-time direct RL estimator is proposed to ascertain the coordinates of each UAV relative to the UGV0 based on intelligent sensing. Secondly, an RL fusion estimation method is proposed to obtain the final estimate of UGV0. Thirdly, an integrated estimation control scheme is also proposed for the application of the RL fusion estimation method to circumnavigation. The convergence and the performance are analyzed. The simulation results validate the effectiveness of the proposed algorithm for RL fusion estimations and of the integrated scheme.

## 1. Introduction

Multi-UAV RL and navigation not only play an increasingly crucial role in civil and military fields [1,2], but have also achieved notable successes in commerce, agriculture, and medical rescue [3,4,5]. Among them, UAV positioning technology [6] is a core element ensuring its safe and efficient operation. By controlling the circumnavigation of UAVs around a central point, the system [7] can achieve precise positioning and navigation in complex environments. In the application of circumnavigation, a multi-UAV RL system [8] must be capable of responding to changing environments and mission requirements in real time. Achieving a good real-time performance may necessitate more complex algorithms and hardware. Therefore, in the design and implementation of a multi-UAV RL system, it is essential to comprehensively address the aforementioned shortcomings and identify corresponding solutions to enhance the robustness and adaptability of the system.

The most common methods include external positioning systems, such as the Global Positioning System (GPS) [9,10] and anchor-based ultra-wideband (UWB) positioning [11,12], which have notably enhanced positioning accuracies. However, in specific environments, such as urban canyons, indoors, or during severe weather conditions, satellite signals may be obstructed or interfered with, thereby limiting the reliability and accuracy of traditional Global Navigation Satellite Systems (GNSSs) [13,14] in these situations. Furthermore, the deployment of an external positioning system can lead to complexities in maintenance and updating. In particular, in systems necessitating long-term operation, the maintenance of both hardware and software can pose challenges.

To address these challenges, researchers are integrating other positioning technologies into UAV systems, including vision sensors [15,16,17]. The integrated use of these technologies enables UAVs to operate in more intricate and demanding environments, accomplishing tasks such as navigating through urban buildings or conducting search and rescue operations in forests. While this approach eliminates the need for infrastructure, visual localization often entails extensive image processing and computational tasks, demanding high-performance hardware and sophisticated algorithms. This can pose challenges in meeting real-time requirements, particularly on resource-constrained UAV platforms.

On the other hand, there are also examples of infrastructure-independent solutions, but they may be inadequate in certain aspects. In [18], a method for RL using radar is proposed. Radar is capable of providing high-precision distance measurements and is very useful for precise RL. However, its equipment cost is high, and it is sensitive to ambient light and transparent objects. Radio Frequency Identification (RFID) systems are suitable for tag recognition at short distances and are applicable for indoor positioning [19]. However, for applications with large-range, high-precision RL requirements, the accuracy of the RFID system may be low. In [20], an RL algorithm based on the fusion of relative navigation sensors was proposed. It mainly involves combining information from different sensors, such as inertial navigation systems and magnetometers, to improve the robustness of RL. Nevertheless, the design and calibration of sensor fusion algorithms are relatively complex and can be susceptible to sensor errors. In [21], an RL algorithm based on visual–inertial navigation fusion was proposed. Combining visual and inertial navigation can overcome the sensor switching problem when transitioning between indoors and outdoors, providing more comprehensive RL information. However, covering large areas may require additional devices to capture enough feature points. The main idea of [22] is to present a weight matrix, simplifying the average consensus algorithm over mobile wireless sensor networks and thereby prolonging the network lifetime as well as ensuring the proper operation of the algorithm. However, the majority of wireless sensor networks discussed in this article are depicted as undirected graphs, which may not adequately address the complexities and fluctuations present in real-world environments.

More recently, a consensus-based leader–follower algorithm was developed in mobile sensor networks, where the goal for the entire network is to converge to the state of the leader [23]. However, this article does not address the positioning issue. A control strategy for a quadrotor elliptical target orbit based on uncertain and non-periodic updates of angle measurements was proposed in [24]. At the translational level, only orientation data are utilized, without incorporating the target’s prior position and velocity information. A position estimator is developed for locating unknown targets. However, the influence of measurement noise is not addressed. In [25], a UAV group circumnavigation control strategy is proposed, in which the UAV circumnavigation orbit is an ellipse whose size can be adjusted arbitrarily. However, the article does not include any research on RL-related aspects. Unlike the work in [26], earlier research focused on utilizing multiple agents to localize targets under fixed topology assumptions. This paper addresses the challenge of cooperative localization in a time-varying topology without an infrastructure, such as anchor points.

In this paper, a directed graph model is employed to represent exchanged information. As measurement failures may occur or a UAV could move beyond the sensing range of its neighbors, the directed graph describing the information flow relationship is time-varying. When a UAV strays beyond the operation area, it triggers a return-to-base protocol, where the UAV autonomously navigates back to the designated area using predefined waypoints or by following a path generated by the control system. To address the RL problem in this scenario, a discrete-time RL direct estimation is proposed for each UAV. The estimation of the relative position concerning its neighbors leverages distance and rate-of-change measurements, the angle of arrival, and the velocities of both itself and its neighbor. The displacements of neighbors during the intervals when distance and rate of change measurements are lost are also taken into account. Moreover, an RL fusion estimation method is devised for each UAV. This fusion estimation involves fusing the relative position estimates of the UAV concerning UGV0 and its neighbors. This approach enables UAVs without direct distance or angle measurements to locate UGV0 with the assistance of their neighbors. Subsequently, we apply the proposed RL fusion estimation algorithm to circumnavigation. In contrast to [25], our system integrates RL fusion estimations with a circumnavigation controller.

The primary contributions are outlined as follows:

(1) When the information flow graph between adjacent UAVs is unidirectional and time-varying, this paper proposes a distributed state observer with state switching to dynamically estimate the positions of UGV0. Only local measurements and limited information exchanges between nearby UAVs are used to estimate the relative coordinates of a group of UAVs concerning a single UGV0. The RL direct estimation error is bounded even in the presence of measurement noise.

(2) To enhance the robustness of RL, consensus-based RL fusion estimation is proposed. The boundedness of the RL fused estimation error is analyzed, and the experimental results demonstrate the effectiveness of the proposed method. The proposed RL method enables each UAV to continuously estimate its relative coordinates to UGV0, even in the absence of any relative measurements concerning UGV0 or its neighbors.

(3) The effectiveness of the entire system was demonstrated through numerical simulations of UAVs using RL fusion estimation for circumnavigation. The system integrates RL into circumnavigation control through UWB ranging and communication networks. The RL scheme proposed in this article applies not only to two-dimensional space but also to three-dimensional space.

The remainder of this article is structured as follows: Section 2 presents the problem formulation. Section 3 proposes an indirect RL method and consistency-based RL fusion estimation. Section 4 discusses the use of RL fusion estimation for circumnavigation. Section 5 conducts simulation experiments, and the article is summarized in Section 6.

## 2. Problem Formulation

**Figure 1:**
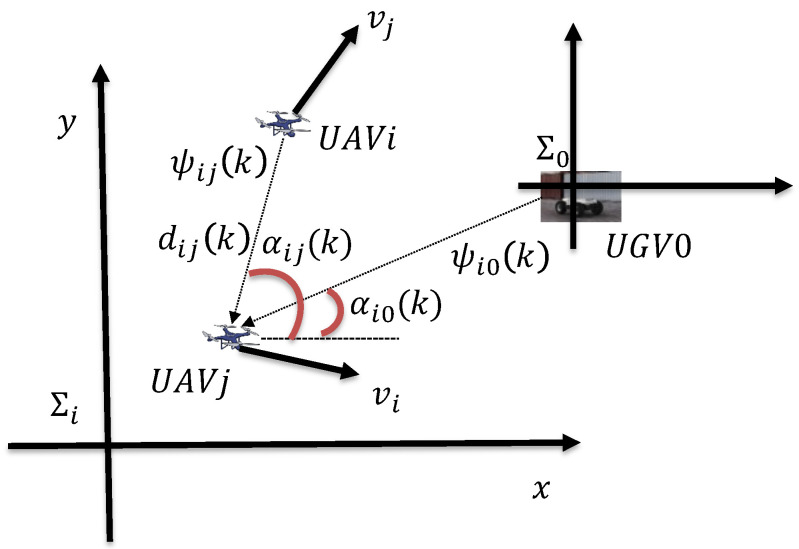
Local measurements and relative positions.

This paper considers a network consisting of a single dynamic UGV and *N* UAVs, labeled 0 and 1,2,…,N, respectively. Define the position of each UAV as $\psi_{i}$. If $j\in \zeta_{i}$, then in the local coordinate system of UAV*i*, the relative position of *j* is $\psi_{ij}=\psi_{j}-\psi_{i}$. Simultaneously, each UAV uses these relative estimates for circumnavigation. Let $\zeta_{i}$ represent the neighbors of UAV or UGV0. When $j\in \zeta_{i}$, UAV*i* can obtain the distance $d_{ij}$ of UAV*j*. Utilizing the obtained angle measurement information $\alpha_{ij}$, UAV*i* can deduce the relative position $\psi_{ij}$ under UAV*j*, as illustrated in Figure 1. Subsequently, leveraging the relative position estimates of its neighbors, each UAV generates corresponding circumnavigation control commands. During circumnavigation, the neighboring UAV*j* is effectively within the sensing radius; i.e., $d_{ij}$ is less than the sensing radius. Assuming a sampling period of *T*, and denoting the sampling time as *k*, for simplicity, *k* is used to represent kT.

Assuming UAVs follow a standard particle model with UAV speeds denoted as $v_{i}$, the relationship between relative speed and position is given by $\psi_{ij}\left(k+1\right)=\psi_{ij}\left(k\right)+Tv_{ij}\left(k\right)$, where $v_{ij}\left(k\right)=v_{j}\left(k\right)-v_{i}\left(k\right)$ represents the relative speed of UAV*j* in the *i* coordinate system. The angle measurement of neighbor *j* is denoted as $\alpha_{ij}\left(k\right)$, and this neighbor can be the target UGV0. It is assumed that each UAV*i* has access to its own speed; distance, represented by $v_{ij}\left(k\right),\;d_{ij}\left(k\right)$; and angle measurement $\alpha_{ij}\left(k\right)$ in its own inertial frame. The reference frame is denoted as $\sum_{i},i=\left\{1,2,\ldots ,N\right\}$ and is the same as $\sum_{0}$. This can be achieved if the UAV is equipped with a compass. Furthermore, assume that each UAV*i* is equipped with airborne sensors, allowing it to obtain the distance measurement ${\dot{\alpha }}_{ij}\left(k\right)$ and change rate ${\dot{d}}_{ij}\left(k\right)$ of its neighbor UAV*j*, or the distance measurement ${\dot{\alpha }}_{i0}\left(k\right)$ and change rate ${\dot{d}}_{i0}\left(k\right)$ of UGV0. As shown in Figure 1, $d_{ij}\left(k\right)=\psi_{ij}\left(k\right)$ and ${\dot{\psi }}_{ij}\left(k\right)=v_{ij}\left(k\right)$ can be obtained. The goal is to develop an estimator so that each UAV can estimate its relative coordinate $\psi_{i0}\left(k\right)$ in UGV0’s frame $\sum_{0}$. With these RL estimates and measurements of distance and orientation between UAVs, the next goal is to integrate RL into circumnavigation control. Next, let us introduce graph theory.

If each UAV is viewed as a node, their interrelationships can be represented by a directed graph denoted as Γ=(u,E), where u={1,2,…,N} is the set of all nodes and *u* corresponds to the set of *N* UAVs. If $j\in \zeta_{i}$, then there is a corresponding arc (i,j)∈E in the directed graph, and UAV*i* can measure the distance, angle, and its corresponding rate of change. To study RL problems (e.g., estimating the position relative to UAV*j*), another weighted directed graph $\ell =(u_{j},E_{j},A)$ is also considered. Here, $u_{j}=\left\{1,2,\ldots ,N\right\}$ is the set of all nodes, $E_{j}\subseteq u_{j}\times u_{j}$ is the set of all arcs in the graph, $A=\left[m_{ij}\right]\in R^{N\times N}$ is the weighted adjacency matrix, and each element of the matrix is positive. Given $i\in u_{j},m_{ii}=0$, if there is an arc (i,j) in graph *ℓ*, then $m_{ij}>0$; otherwise, $m_{ij}=0$. It is worth noting that *ℓ* may be time-varying due to possible interruptions in the measurement. Assume that the set Γ comprises all ordinary UAVs, referred to as the original set. UGV0 is introduced as the source point to form a new set $\bar{\Gamma }=\left(\bar{u},\bar{E}\right)$, known as the expanded set. Here, $\bar{u}=\left\{0\right\}\cup u,\;\bar{E}=E_{0}\cup E$, where $E_{0}$ denotes the edge set comprising UGV0 and its surrounding neighbor UAVs. $\left(i,j\right)\in \bar{E}$ signifies that UAV*i* and UAV*j* can exchange speed and data packets.

Define the Laplacian matrix of the weighted directed graph *ℓ* as $L_{\ell }$, and the diagonal matrix $P=\mathrm{diag}\left\{p_{1},p_{2},\ldots ,p_{N}\right\}\in R^{N\times N}$ as the degree matrix of *ℓ*, where the diagonal element is $p_{i}=\sum_{j\in \zeta_{i}}m_{ij},i=\left\{1,2,\ldots ,N\right\}$. To investigate the RL problem, a system comprising *N* UAVs and UGV0 is associated with another graph. The graph *ℓ* composed of *N* UAVs is a subgraph of $\bar{\Gamma }$. Let ${\bar{\zeta }}_{i}$ represent the set of neighbor nodes of node *i* in $\bar{\Gamma }$, which may include UGV0. If UAV*i* can obtain direct observation of the UGV0 distance $d_{i0}\left(k\right)$ or angle measurement $\alpha_{i0}\left(k\right)$, then $0\in \bar{\zeta_{i}}$; otherwise, $0\notin \bar{\zeta_{i}}$. Define a diagonal matrix $\beta \in R^{N\times N}$ as the target adjacency matrix associated with $\bar{\Gamma }$, with diagonal element $s_{i},\;i=\left\{1,2,\ldots ,N\right\}$. If UAV*j* is the neighbor of UAV*i*, and UAV*i* can directly obtain the distance and angle measurement of UAV*j*, then $s_{i}>0$; otherwise, $s_{i}=0$. For $\bar{\Gamma }$, if there exists a pathway from UGV0 to UAV*i*, we consider UGV0 to be jointly reachable.

## 3. Cooperative RL Algorithm

In this section, we propose a distributed RL algorithm based on mixed measurements. It addresses the challenge of estimating the relative coordinates of a UAV in the local frame of its neighbors. Through this approach, if UGV0 is a neighbor of the UAV, the UAV can acquire relative measurements of UGV0. Subsequently, the UAV can directly estimate its coordinates in the local frame of UGV0, termed a direct estimate. Conversely, if UGV0 is not a neighbor of the UAV, the UAV cannot utilize the relative measurements between UGV0 and itself to estimate its coordinates concerning UGV0. In this scenario, if the UAV can access the relative coordinates of its neighbors and the neighbors know the relative coordinates of UGV0, the UAV can deduce the relative coordinates of UGV0 through its neighbors. Nevertheless, multiple neighbors could potentially aid the UAV in establishing the relative coordinates of UGV0 through this method. To avoid dependence on unique neighbors, it is essential to combine multiple estimates. Furthermore, even with the availability of direct estimation, combining indirect estimation can enhance accuracy and expedite convergence. Hence, a consensus-based fusion estimation method is devised for each UAV in the second part of this section to fuse both direct estimates and all accessible indirect estimates.

### 3.1. RL Direct Estimation Relies on Persistent Excitation

As we all know, UAVs can experience measurement and communication losses due to harsh environments or sensor failures. In this subsection, we assume that UAV*i* (i=1,2,…,N) can communicate with UGV0 and has access to distance measurement $d_{i0}\left(k\right)$ and the rate of change ${\dot{d}}_{i0}\left(k\right)$ in certain time intervals, in addition to angle measurement $\alpha_{i0}\left(k\right)$ and ${\dot{\alpha }}_{i0}\left(k\right)$. A direct estimator is designed to estimate the relative coordinates $\psi_{ij}\left(k\right)$ of UAV*i* in the local frame of UAV*j*.

**Figure 2:**
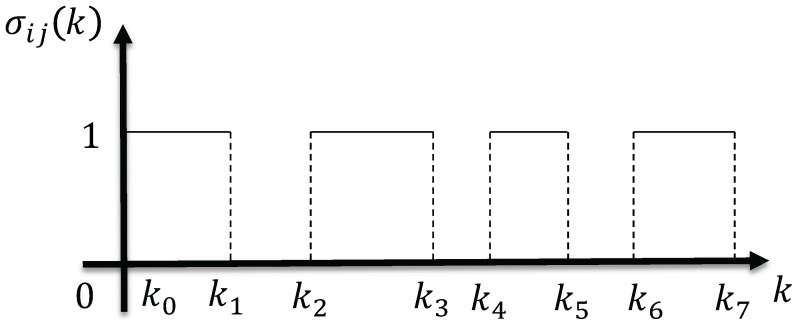
Diagram illustrating the indicator function.

Due to unreliability in local relative measurements, assume that UAV*i* obtains measurements relative to UAV*j*, $d_{ij}\left(k\right)$ and ${\dot{d}}_{ij}\left(k\right)$, or angle measurements $\alpha_{ij}\left(k\right)$ and ${\dot{\alpha }}_{ij}\left(k\right)$ at time $k\in \left[k_{0},k_{1}\right)\cup \left[k_{2},k_{3}\right)\cup \ldots$, with a measurement break at $k\in \left[k_{0},k_{1}\right)\cup \left[k_{2},k_{3}\right)\cup \ldots$. An indicator function, denoted as $\sigma_{ij}\left(k\right)$, is defined to represent the status. When $\sigma_{ij}\left(k\right)=1$, UAV*i* can acquire local relative measurements $d_{ij}\left(k\right)$ and ${\dot{d}}_{ij}\left(k\right)$ or angle measurement $\alpha_{ij}\left(k\right)$ and ${\dot{\alpha }}_{ij}\left(k\right)$; otherwise, $\sigma_{ij}\left(k\right)=0$. As illustrated in Figure 2, $\left.\sigma_{ij}\left(k\right)=\left\{\begin{array}{cc} 1,k\in [k_{2t},k_{2t+1}),\\ 0,k\in [k_{2t+1},k_{2t+2}), & t=0,1,\ldots \end{array}\right.\right.$. Taking the derivatives on both sides of $d_{ij}^{2}\left(k\right)={∥\psi_{ij}\left(k\right)∥}^{2},$ we can obtain $d_{ij}\left(k\right){\dot{d}}_{ij}\left(k\right)=v_{ij}^{T}\left(k\right)\psi_{ij}\left(k\right)$. Two unit vectors are constructed from the angle measurement information $\alpha_{ij}\left(k\right)$: the unit vector $\Phi_{ij}\left(k\right)={\left[\begin{array}{cc} \cos\alpha_{ij}\left(k\right) & \sin\alpha_{ij}\left(k\right) \end{array}\right]}^{T}$ pointing from UAV*i* to UAV*j*, and the vector $\Psi_{ij}\left(k\right)={\left[\begin{array}{cc} -\sin\alpha_{ij}\left(k\right) & \cos\alpha_{ij}\left(k\right) \end{array}\right]}^{T}$ obtained by rotating it 90° counterclockwise. Because vectors $\Phi_{ij}\left(k\right)$ and $\psi_{ij}\left(k\right)$ have the same direction, and $\Phi_{ij}\left(k\right)$ and $\Psi_{ij}\left(k\right)$ are perpendicular to each other, the constraint equation $\Psi_{ij}{\left(k\right)}^{T}\psi_{ij}\left(k\right)=0$ can be obtained. When $\sigma_{ij}\left(k\right)=1$, UAV*i* can obtain the estimation algorithm of ${\hat{\psi }}_{ij}\left(k\right)$ through information from measurements and communication. Estimates can become inaccurate due to UAV motion, as the sensors are subject to interference. Assume that the estimated value ${\hat{\psi }}_{ij}\left(k\right)$ before the interruption is initially employed. Once communication and measurement are restored, that is, when $\sigma_{ij}\left(k\right)=1$, UAV*i* will correct the deviation caused by the estimated value in the case of $\sigma_{ij}\left(k\right)=0$. Considering sensor noise, the RL direct estimation of UAV*i* in the local coordinate system of UAV*j* at time *k* is estimated as follows:

$${\hat{\psi }}_{ij}\left(k+1\right)={\hat{\psi }}_{ij}\left(k\right)+T\left(v_{ij}\left(k\right)+\tau \left(k\right)\right)\left[K{\hat{\Upsilon }}_{ij}\left(k\right)-{\left(v_{ij}\left(k\right)+\tau \left(k\right)\right)}^{T}{\hat{\psi }}_{ij}\left(k\right)\right]$$

(1) $$\left.\left\{\begin{array}{cc} {\hat{\Upsilon }}_{ij}\left(k\right)=\left(d_{ij}\left(k\right)+\sigma \left(k\right)\right)\left({\dot{d}}_{ij}\left(k\right)+\dot{\sigma }\left(k\right)\right) & \delta =1\\ {\hat{\Upsilon }}_{ij}\left(k\right)=\frac{{\left(v_{ij}\left(k\right)+\tau \left(k\right)\right)}^{2}\cos\left(\alpha_{ij}\left(k\right)+\Delta \left(k\right)\right)\sin\left(\alpha_{ij}\left(k\right)+\Delta \left(k\right)\right)}{\left({\dot{\alpha }}_{ij}\left(k\right)+\dot{\Delta }\left(k\right)\right)+{\dot{\theta }}_{ij}}\cdot & \delta =0 \end{array}\right.\right.k\in \left[k_{2t},k_{2t+1}\right)$$

$${\hat{\psi }}_{ij}\left(k+1\right)={\hat{\psi }}_{ij}\left(k\right)\;k\in \left[k_{2t+1},k_{2t+2}\right).$$

where $\tau \left(k\right),\;\sigma \left(k\right),\;\dot{\sigma }\left(k\right),\;\Delta \left(k\right),\;\dot{\Delta }\left(k\right)$ represent the measurement noise of $v_{ij}\left(k\right),\;d_{ij}\left(k\right),\;{\dot{d}}_{ij}\left(k\right),\;\alpha_{ij}\left(k\right),\;{\dot{\alpha }}_{ij}\left(k\right)$ at time *k*, respectively. *K* is a tunable constant gain. ${\hat{\psi }}_{ij}\left(k\right)$ represents the coordinate of UAV*i* in the local coordinate system $\sum_{j}^{M}$ of UAV*j*. When δ=1, it indicates the distance measurement is normal, and when δ=0, it indicates the angle measurement is normal. It will be demonstrated later that the RL direct estimation error is bounded in the presence of noise.

Let the estimation error of the above observer (1) be denoted as ${\tilde{\psi }}_{ij}\left(k\right)={\hat{\psi }}_{ij}\left(k\right)-\psi_{ij}\left(k\right)$, and the dynamic equation of the estimation error is

(2) $${\tilde{\psi }}_{ij}\left(k+1\right)=\left\{\begin{array}{cc} \left[I-T\left(v_{ij}\left(k\right)+\tau \left(k\right)\right){\left(v_{ij}\left(k\right)+\tau \left(k\right)\right)}^{T}\right]{\tilde{\psi }}_{ij}\left(k\right) & k\in [k_{2t},k_{2t+1})\\ {\tilde{\psi }}_{ij}\left(k\right)+T\left(v_{ij}\left(k\right)+\tau \left(k\right)\right) & k\in [k_{2t+1},k_{2t+2}) \end{array}\right..$$

Let us begin by introducing the concept of persistent excitation [27] before moving on to the discussion of the convergence of error system (2). There exists a positive integer *m*, $\alpha_{1},\alpha_{2}$ such that for any $k\in Z^{+}$, there is

(3) $$\alpha_{1}I\leq \sum_{f=k}^{k+m}\left(v_{ij}\left(k\right)+\tau \left(k\right)\right){\left(v_{ij}\left(k\right)+\tau \left(k\right)\right)}^{T}T\leq \alpha_{2}I,$$

where *T* is the sampling period. Next, let us analyze the physical meaning of persistent excitation. Assuming that the speed of each UAV is continuously differentiable and bounded, the upper bound obviously holds. Therefore, the main focus is on (3), the lower bound. Expanding Equation (3), we can obtain

$$\begin{array}{cc} & \sum_{f=k}^{k+m}\left(v_{ij}\left(f\right)+\tau \left(f\right)\right){\left(v_{ij}\left(f\right)+\tau \left(f\right)\right)}^{T}=\\ & \left[\begin{array}{cc} \sum_{f=k}^{k+m}{\left(v_{ijx}\left(f\right)+\tau \left(f\right)\right)}^{2} & \sum_{f=k}^{k+m}\left(v_{ijx}\left(f\right)+\tau \left(f\right)\right)\left(v_{ijy}\left(f\right)+\tau \left(f\right)\right)\\ \sum_{f=k}^{k+m}\left(v_{ijx}\left(f\right)+\tau \left(f\right)\right)\left(v_{ijy}\left(f\right)+\tau \left(f\right)\right) & \sum_{f=k}^{k+m}{\left(v_{ijy}\left(k\right)+\tau \left(f\right)\right)}^{2} \end{array}\right] \end{array}.$$

The two components of $v_{ij}\left(k\right)$ are represented as $v_{ijx}\left(k\right)$ and $v_{ijy}\left(k\right)$, which are linearly independent of f∈{k,…,k+m}. According to the inequality of Cauchy–Bunyakovsky, for any k≥0, the following formula

$$\begin{array}{c} {\left(\sum_{f=k}^{k+m}\left(v_{ijx}\left(f\right)+\tau \left(f\right)\right)\left(v_{ijy}\left(f\right)+\tau \left(f\right)\right)\right)}^{2}\leq \sum_{f=k}^{k+m}{\left(v_{ijx}\left(f\right)+\tau \left(f\right)\right)}^{2}\sum_{f=k}^{k+m}{\left(v_{ijy}\left(f\right)+\tau \left(f\right)\right)}^{2} \end{array}$$

holds. Both sides of the above inequality are equal if and only if $v_{ijx}\left(f\right)$ and $v_{ijy}\left(f\right)$ are linearly related.

**Theorem** **1.** *According to Equation (1), when persistent excitation (3) holds for UAVi and if the sampling period T satisfies the given criterion*

(4) $$0<T<\frac{2(\bar{v}+\bar{\tau })}{K^{2}}$$

*then the estimation error of system (2) is bounded. The upper bound $v_{i}$ of the error is determined by a specific value $\bar{v}$, ensuring that the error satisfies $0<∥v_{ij}∥\leq 2\bar{v}$, the specified condition. To proceed, suppose there exists a constant $\bar{\tau }>0$ such that $∥\tau \left(k\right)∥\leq \bar{\tau }.$*

**Proof.**  Firstly, consider the system related to (2):

(5) $$\varepsilon \left(k+1\right)=\left[I-TK\sigma_{ij}\left(k\right)\left(v_{ij}\left(k\right)+\tau \left(k\right)\right){\left(v_{ij}\left(k\right)+\tau \left(k\right)\right)}^{T}\right]{\tilde{\psi }}_{ij}\left(k\right).$$

Construct the Lyapunov function $V\left({\tilde{\psi }}_{ij}\left(k\right)\right)={\tilde{\psi }}_{ij}\left(k\right){\tilde{\psi }}_{ij}^{T}\left(k\right)$, and the difference in the Lyapunov function within *m* time steps can be expressed as

(6) $$\begin{array}{cc} \Delta V_{m}\left(k\right) & =V\left({\tilde{\psi }}_{ij}\left(k+m\right)\right)-V\left({\tilde{\psi }}_{ij}\left(k\right)\right)\\ & =\sum_{f=k}^{k+m}C\left(k\right){\left({\left(v_{ij}\left(k\right)+\tau \left(k\right)\right)}^{T}{\tilde{\psi }}_{ij}\left(k\right)\right)}^{2}. \end{array}$$

In addition, applying the induction method to system (5), the *f*-step RL direct estimation error range $∥{\tilde{\psi }}_{ij}\left(f\right)∥\leq ∥{\tilde{\psi }}_{ij}\left(k\right)∥+\tau \left(f\right)T\left(1+K\left(2\bar{v}+\bar{\tau }\right)\right)\left(f-k\right)$ is obtained. Now, (6) can be expressed as

(7) $$\begin{array}{cc} \Delta V_{m}\left(k\right) & \leq \sum_{f=k}^{k+m}C(k)\left(v_{ij}\left(f\right)+\tau \left(f\right)\right){\left(v_{ij}\left(f\right)+\tau \left(f\right)\right)}^{T}\\ & +m\tau \left(f\right)T\left(1+K(2\bar{v}+\tau \left(f\right))\right)\left(2∥{\tilde{\psi }}_{ij}\left(k\right)∥+m\tau \left(f\right)T\left(1+K(2\bar{v}+\bar{\tau })\right)\right). \end{array}$$

Observing (2) and (5), we can see that if ${\tilde{\psi }}_{ij}\left(k\right)=\varepsilon \left(k\right)$, then for any $k\in [k_{2t},k_{2t+1}),$ ${\tilde{\psi }}_{ij}\left(k_{2t}\right)=\varepsilon \left(k_{2t}\right)$ is satisfied. In the $k\in [k_{2t},k_{2t+1})$ interval, ε(k) remains unchanged, while ${\tilde{\psi }}_{ij}\left(k\right)$ is time-varying. However, it can be seen from (2) that when $k=k_{2t+2}$, ${\tilde{\psi }}_{ij}\left(k_{2t+2}\right)={\tilde{\psi }}_{ij}\left(k_{2t+1}\right)$. To sum up, it can be deduced that as long as ${\tilde{\psi }}_{ij}\left(0\right)=\varepsilon \left(0\right)$, then for any $k\in [k_{2t}$, $k_{2t+1}),{\tilde{\psi }}_{ij}\left(k\right)=\varepsilon \left(k\right)$.

In addition, according to the definition of $\sigma_{ij}\left(k\right)$, when $k\in [k_{2t}$, $k_{2t+1}),\;\sigma_{ij}\left(k\right){\tilde{\psi }}_{ij}\left(k\right)={\tilde{\psi }}_{ij}\left(k\right)$. When $k\in [k_{2t+1}$, $k_{2t+2}),\;\sigma_{ij}\left(k\right){\tilde{\psi }}_{ij}\left(k\right)=0$. It can be seen from (3) that the RL direct estimation error of (5) is bounded in the case of noise interference. That is, there exists α>0, C(k)>0 that satisfies for any $k\geq 0,\;∥\varepsilon \left(k\right)∥\leq C\left(k\right)e^{-\alpha k}$, if and only if there exists $\alpha_{1}>0,$ $\alpha_{2}>0,\;T>0$, and (3) holds for any k≥0. To now, the RL direct estimation error bound can be expressed as follows. Combining (4) and (7) leads to $\Delta V_{m}\left(k\right)\leq \sigma_{ij}\left(k\right){\tilde{\psi }}_{ij}\left(k\right)+\alpha_{1}C\left(k\right)e^{-\alpha k}+\alpha_{2}$, where $\sigma_{ij}\left(k\right)>0,\;\alpha >0,\;C\left(k\right)>0$. Therefore, when $k\in \left[k_{2t+1},k_{2t+2}\right),\;{\tilde{\psi }}_{ij}\left(k\right)\leq \alpha_{1}C\left(k\right)e^{-\alpha k}+\alpha_{2}$. It can be proven that when $k\in \left[k_{2t},k_{2t+1}\right),\;{\tilde{\psi }}_{ij}\left(k\right)\leq \left(1+\alpha_{1}\right)C\left(k\right)e^{-\alpha k}+\alpha_{2}$; the proof of Theorem 1 is complete. □

### 3.2. Fusion-Based RL Estimation

In the previous Section 3.1, we assumed that local relative measurements ($d_{ij}\left(k\right)$, rate of change ${\dot{d}}_{ij}\left(k\right)$, and $\alpha_{ij}\left(k\right),{\dot{\alpha }}_{ij}\left(k\right)$) are unreliable, with measurement values available only at certain moments. An estimator was then designed for the UAV*i* to position UGV0. If UGV0 is a neighbor of UAV*i*, then UAV*i* can obtain its direct estimate ${\hat{\psi }}_{i0}\left(k\right)$ in the local coordinate system of UGV0. However, due to harsh environments or temporary sensor failures, a UAV may lose its local relative measurements. Worse still, some UAVs may not always have relative measurements with respect to UGV0 because UGV0 is outside their sensing range. In this case, cooperation among neighbor UAVs is needed to help UGV0 localize. In this subsection, an RL indirect estimation method is developed for each UAV*i* to estimate its relative coordinates with respect to UAV*j*, even when it may lack direct measurements for UGV0 or experience measurement failures over time.

**Figure 3:**
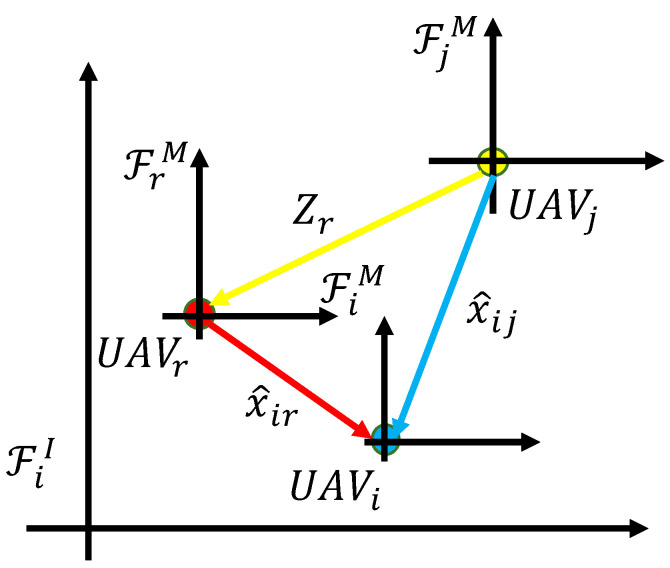
Obtain the indirect RL estimate ${\hat{x}}_{ij}\left(k\right)$ from UAV*i* to UAV*j* through UAV*r*.

If UAV*i* can estimate its coordinate ${\hat{x}}_{ir}$ relative to neighboring UAV*r*, UAV*r* can also share its estimate $Z_{r}$ with UAV*i*. As a result, UAV*i* can indirectly obtain its estimate relative to UAV*j* through UAV*r*, as illustrated in Figure 3. The formula is as follows:

(8) $${\hat{x}}_{ij}\left(k\right)={\hat{x}}_{ir}\left(k\right)+Z_{r}\left(k\right).$$

Expanding on both direct and indirect RL algorithms, we also explore RL fusion estimation between UAVs to achieve target positioning. RL fusion estimation serves a dual purpose. It aids UAVs lacking direct target measurements, enhancing their ability to locate targets. Simultaneously, it bolsters the robustness of relying on RL direct estimation. Utilizing information gathered by UAVs and integrating both direct (1) and indirect (8) RL estimators, an RL fusion estimation method is proposed. Each UAV*i* employs the following estimation method to update its final estimate, as expressed in the formula:

(9) $$\begin{array}{cc} Z_{i}\left(k+1\right) & =Z_{i}\left(k\right)+T\left(v_{ij}\left(k\right)+\tau \left(k\right)\right)\\ & +\sum_{j\in \zeta_{i}\left(k\right)}\beta_{ij}\left(k\right)\left[{\hat{x}}_{i0}\left(k\right)-Z_{i}\left(k\right)\right]. \end{array}$$

Consequently, each UAV*i* updates its fused estimate using (9), irrespective of its ability to directly obtain relative measurements about UGV0.

**Theorem** **2.** *If the conditions of Theorem 1 are met; if it is assumed that, for every node pair (i,j) in Γ(k) that appears infinitely many times, the persistent excitation (3) is satisfied; if each node in Γ(k) is uniformly jointly reachable from UGV0; and if the fusion weight satisfies $0<\sum_{j\in \zeta_{i}\left(t\right)}\beta_{ij}\left(k\right)<1$, then the fusion estimate $Z_{i}\left(k\right)$ of each UAVi asymptotically converges to its true coordinates. In the presence of measurement noise, the RL fused estimation error is bounded.*

**Definition** **1** (Stability of a discrete system input state [28]). *Consider a nonlinear system given by equation x(k+1)=f(x(k),u(k)). If there exists a function class KL: $\beta :R_{\geq 0}\times R_{\geq 0}\to R_{\geq 0}$ and a classK function γ such that for any control input $u\in l_{\infty }^{m}$ and any $\xi \in R^{n}$, the following formula |x(k,ξ,u)|≤β(|ξ|,k)+γ(∥u∥) holds for any positive integer k, then the system in Definition 1 is said to be input-to-state stable (ISS).*

**Lemma** **1** ([28]). *If A is a Schur matrix, then the discrete system x(k+1)=Ax(k)+Bu(k) is ISS*.

**Lemma** **2** ([29]). *Matrix $L_{j}+B_{j}$ is positively stable, indicated by eigenvalues with positive real parts, if and only if UAVj is jointly reachable in* Γ.

**Proof.**  For any given i={1,2,…,N}, let $y_{i}\left(k\right)=Z_{i}\left(k\right)-\psi_{ij}\left(k\right)$. Then, (9) can be rewritten as

(10) $$y_{i}\left(k+1\right)=y_{i}\left(k\right)+\sum_{j\in \zeta_{i}\left(k\right)}\beta_{ij}\left(k\right)\left[y_{j}\left(k\right)-y_{i}\left(k\right)\right]+u_{ij}\left(k\right),$$

where the $u_{ij}\left(k\right)=\sum_{j\in \zeta_{i}\left(t\right)}\beta_{ij}\left(k\right){\tilde{\psi }}_{ij}\left(k\right)+T\tau \left(k\right)$ part serves as the input signal. Aggregating all the equations in i={0,1,2,…,N}, (10) can be expressed in matrix form as

(11) $$y_{ij}\left(k+1\right)=\left(\left(I-L_{j}-B_{j}\right)⊗I_{p}\right)y_{ij}\left(k\right)+u_{ij}\left(k\right),$$

where $y_{ij}\left(k\right)={\left[y_{0}^{T}\left(k\right)y_{1}^{T}\left(k\right)\ldots y_{N}^{T}\left(k\right)\right]}^{T},\;u_{ij}\left(k\right)={\left[u_{0}^{T}\left(k\right)u_{1}^{T}\left(k\right)\ldots u_{N}^{T}\left(k\right)\right]}^{T}.$*p* is two or three, determined by the dimensions of the environmental space. Lj represents the weighted Laplacian matrix of the graph Γ, and Bj is the adjacency matrix of *j* associated with Γ.

Firstly, considering the unforced system in

(12) $$y_{ij}\left(k+1\right)=\left(\left(I-L_{j}-B_{j}\right)⊗I_{p}\right)y_{ij}\left(k\right),$$

it can be verified that all eigenvalues of $L_{j}+B_{j}$ strictly lie within the unit circle centered at the origin. Therefore, $L_{j}+B_{j}$ is a Schur matrix. As UAV*i* and UGV0 are uniformly and jointly reachable, according to Lemmas 1 and 2, when k→∞, all components of the solution of (12) uniformly converge exponentially to a certain common value. It can be concluded that when $k\to \infty ,\;y_{i}\left(k\right)\to c$ (*c* is a constant), indicating the exponential stability of (12).

Consider (11) next. According to Definition 1, for any $k\geq 0,\;∥y_{ij}\left(k\right)\left(y_{ij}\left(0\right),u_{ij}\left(k\right)\right)∥\leq \beta (∥y_{ij}\left(0\right)∥,k)+\gamma (∥u_{ij}\left(k\right)∥)$. According to Theorem 1, for any positive constant $\beta_{ij}\left(k\right),$ $∥u_{ij}\left(k\right)∥\leq \sum_{j\in \zeta_{i}\left(k\right)}\beta_{ij}\left(k\right)+T\tau \left(k\right)$. Since γ(·) is a function of classK, it follows that when $k\to \infty ,\;\gamma (∥u_{ij}\left(k\right)∥)\to c$. Because $y_{ij}\left(0\right)$ is bounded and β(·,·) is a function of classKL, when $k\to \infty ,\;\beta (∥y_{ij}\left(0\right)∥,k)\to 0$. Therefore, $∥y_{ij}\left(k\right)\left(y_{ij}\left(0\right),u_{ij}\left(k\right)\right)∥\to c$ when k→∞. The proof of Theorem 2 is complete. □

## 4. Integrated Solutions for RL and Circumnavigation

**Figure 4:**
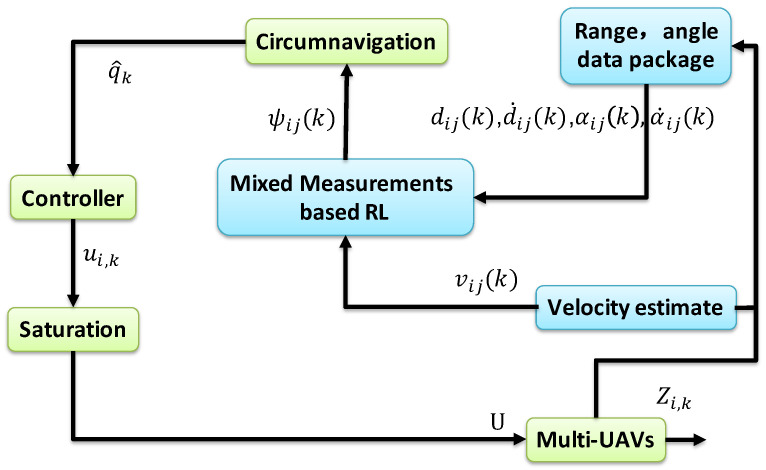
An integrated RL and circumnavigation solution.

In this section, we propose an integrated solution combining RL and circumnavigation to facilitate the rotation of multi-UAVs around UGV0 while maintaining a circular formation. This capability is particularly valuable in practical scenarios like surrounding and entrapping a hostile target. As depicted in Figure 4, it relies on distance $d_{i}\left(k\right)=∥Z_{i}\left(k\right)-p^{*}∥$ and angle measurements $\alpha_{ij}\left(k\right)$. An adaptive estimator will be formulated to attain relative positioning with the assistance of a specifically designed bounded input $u_{i}\left(k\right)$. Let $q_{i}\left(k\right)=Z_{i}\left(k\right)-p^{*}$ denote the relative position to the UGV0. ${\hat{q}}_{i}\left(k\right)$ is expressed as an estimate of $q_{i}\left(k\right)$. Note that $u_{i}\left(k\right)$ should also satisfy $∥u_{i}\left(k\right)∥\leq U$ to address the circumnavigation problem (13), where *U* is the maximum velocity.

Given UGV0 at any location $p^{*}\left(k\right)$, the objective is to enable the UAV to orbit around UGV0. The formula is as follows:

(13) $$\lim_{k\to \infty }Z_{i}\left(k\right)=p^{*}\left(k\right),$$

where $Z_{i}\left(k\right)$ represents the position of UAV*i* at time step *k*. For trajectory planning purposes, consider a discrete-time integrator model with bounded velocity: $Z_{i}\left(k+1\right)=Z_{i}\left(k\right)+Tu_{i}\left(k\right),$ where *T* is the sampling period.

Based on this, a circumnavigation control law involving RL fusion estimation is proposed:

(14) $$u_{i,k}=\left\{\begin{array}{cc} u_{0,k}-\left[\left({\hat{d}}_{i}^{2}\left(k\right)-d_{i}^{2}\left(k\right)\right)I-\beta E\right]{\hat{q}}_{i}\left(k\right) & \delta =1\\ \left({\hat{d}}_{i}\left(k\right)-d_{i}\left(k\right)\right)\Phi_{ij}\left(k\right)+\alpha \Psi_{ij}\left(k\right) & \delta =0 \end{array}\right.,$$

where β is any non-zero real scalar and *E* is the rotation matrix. ${\hat{d}}_{i}\left(k\right)$ represents the estimated value of $d_{i}\left(k\right)$. Additionally, a constant positive scalar α is introduced. By integrating the previously developed RL algorithm (9) with the circumnavigation control algorithm (14), we propose an integrated RL fusion estimation and circumnavigation control algorithm.

## 5. Simulation

**Figure 5:**
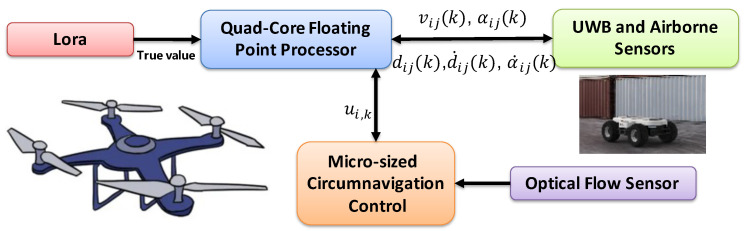
Workflow diagram for circumnavigation control.

In this part, we consider a cooperative RL fusion estimation involving five mobile UAVs and a slow UGV0 and subsequently apply the estimated values to circumnavigation control. The simulation workflow diagram is illustrated in Figure 5.

### 5.1. Results of RL Fusion Estimation Simulations

**Figure 6:**
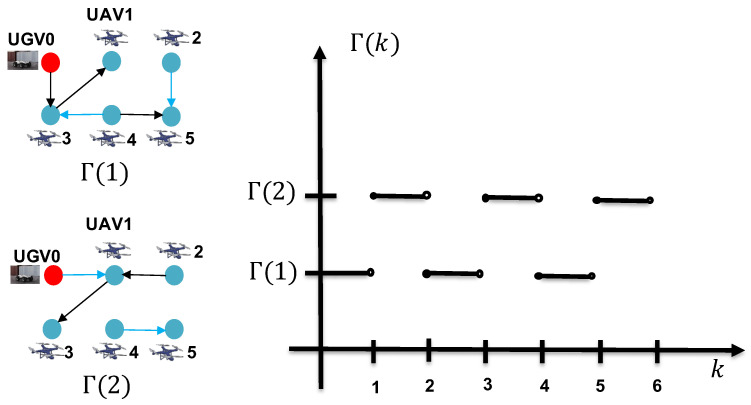
The periodic switching graph Γ(k) that switches between two different topologies Γ(1) and Γ(2).

**Figure 7:**
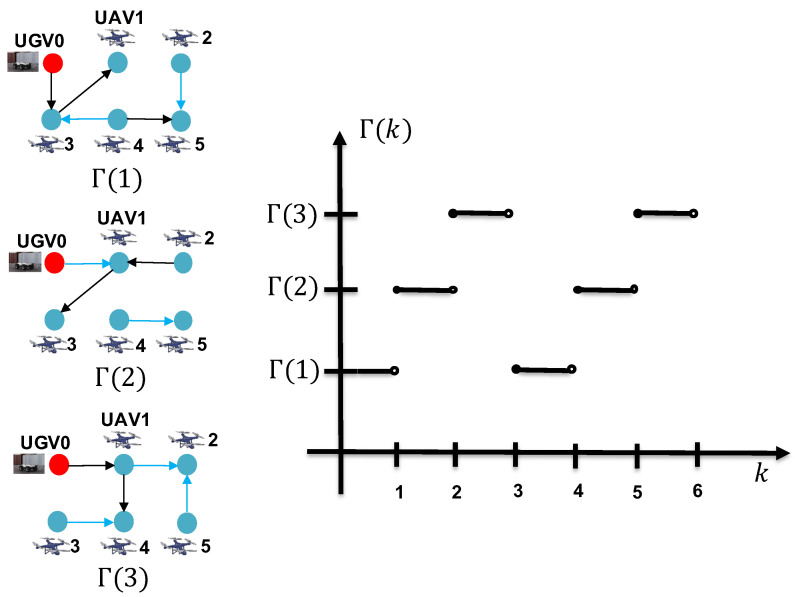
The three graphs Γ(1), Γ(2), and Γ(3), among which Γ(k) randomly switches.

In this section, a simulation of the RL of five mobile UAVs and a slowly moving UGV0 in two-dimensional space is conducted. The information flow graph Γ(k) between the five UAVs and UGV0 is allowed to switch between two graphs periodically, Γ(1) and Γ(2), as illustrated in Figure 6, or switch between three graphs randomly, Γ(1), Γ(2), and Γ(3), as illustrated in Figure 7. It can be confirmed that in the information flow graph, UGV0 is jointly reachable. UAV 1 and 3 periodically acquire direct measurements of UGV0, while UAV 2, 4, and 5 do not; they rely solely on indirect estimates through their neighbors. To validate the proposed estimation scheme, the target UGV0 is positioned at the origin, and the five UAVs are subjected to the following dynamic control:

$$\begin{array}{cc} {\dot{x}}_{1}\left(k\right) & =\left[\begin{array}{c} -\sin k\\ \cos k \end{array}\right],\;{\dot{x}}_{2}\left(k\right)=\left[\begin{array}{c} 0\\ \frac{1}{4} \end{array}\right]\\ {\dot{x}}_{3}\left(k\right) & =\left[\begin{array}{c} \frac{1}{6}\\ 0 \end{array}\right],\;{\dot{x}}_{4}\left(k\right)=\left[\begin{array}{c} 0\\ \frac{1}{8} \end{array}\right],\;{\dot{x}}_{5}\left(k\right)=\left[\begin{array}{c} -2\sin k\\ 2\cos k \end{array}\right]. \end{array}$$

Furthermore, the initial positions of the five UAVs are labeled as (−1, −2), (6, 8), (8, 9), (11, −2), and (1, 9), respectively. The initial position of UGV0 is (0.3, 0).

**Figure 8:**
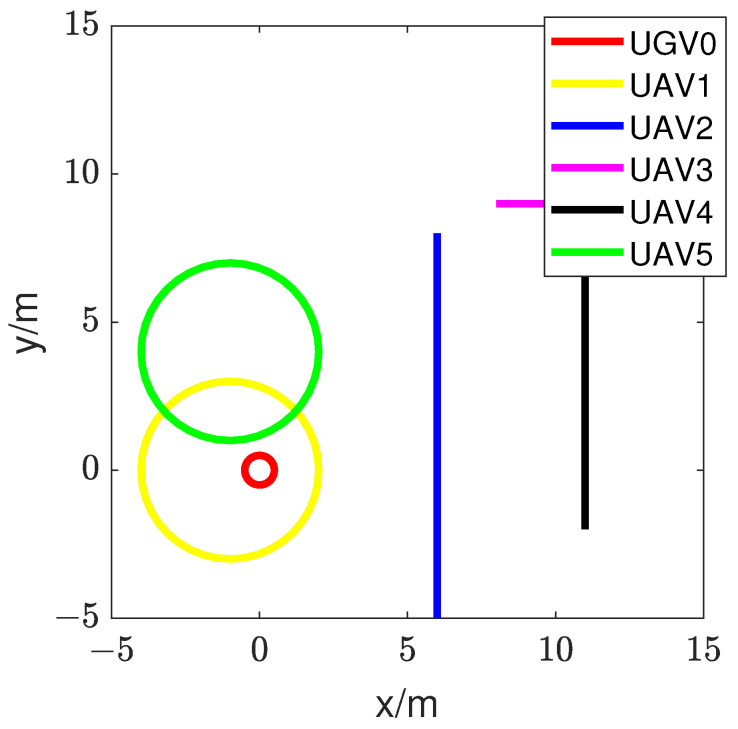
Movement trajectories of the five UAVs and UGV0.

To validate the feasibility of the proposed RL fusion estimation scheme, we designated UGV0 as the relative target for estimation. In the first simulation, the trajectory diagram is depicted in Figure 8. In the second simulation, we let all of the UAVs move randomly within the range [−50, 50]. Communication protocols were used to ensure that UAVs were aware of each other’s positions and velocities, allowing them to maintain safe distances. Upon receiving position and velocity updates from neighboring UAVs, each UAV processes this information to determine the relative positions and velocities of nearby UAVs. By comparing this information with its trajectory, a UAV can assess the risk of potential collisions and take appropriate action to avoid them. With unlimited energy, UAVs can perform their tasks without the need to consider energy efficiency or battery life. They can fly for extended periods, cover long distances, and execute complex maneuvers without the risk of power depletion.

**Figure 9:**
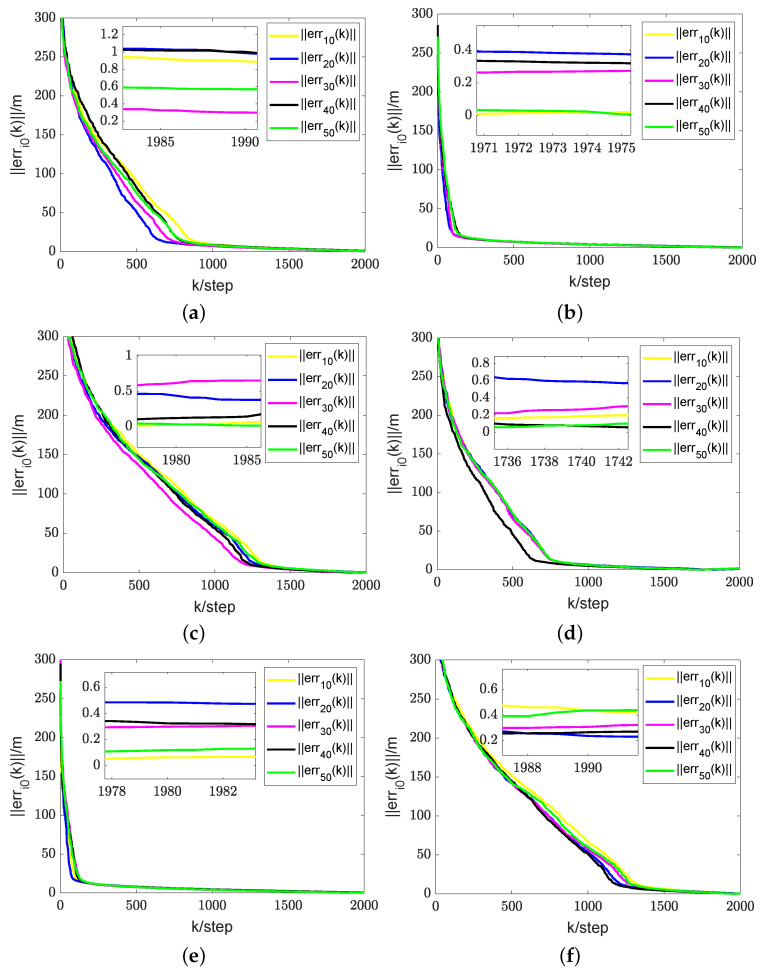
The first simulation. (**a**) Results of RL fusion estimation errors under periodic switching conditions using the method in [30]. (**b**) Results of RL fusion estimation errors under periodic switching conditions using the proposed method. (**c**) Results of RL fusion estimation errors under periodic switching conditions using the method in [31]. (**d**) Results of RL fusion estimation errors under random switching conditions using the method in [30]. (**e**) Results of RL fusion estimation errors under random switching conditions using the proposed method. (**f**) Results of RL fusion estimation errors under random switching conditions using the method in [31].

**Figure 10:**
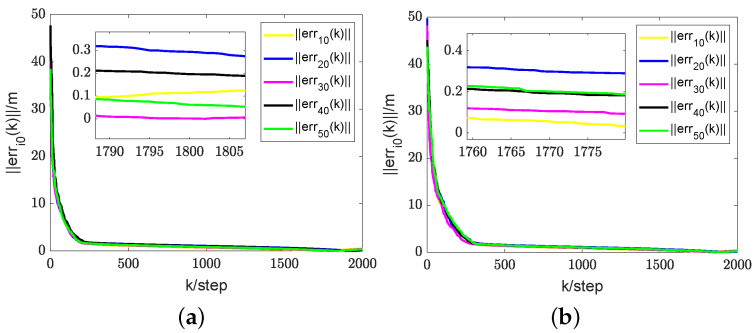
The second simulation. (**a**) Results of RL fusion estimation errors under periodic switching conditions using the proposed method. (**b**) Results of RL fusion estimation errors under random switching conditions using the proposed method.

For the first simulation, it can be verified that, in the case of a time-varying information flow graph, each UAV is uniformly jointly reachable to UGV0. Additionally, it can be confirmed that the persistent excitation (3) is satisfied. For the second simulation, it is challenging to strictly confirm the condition that each UAV is consistently jointly reachable to UGV0 and that the persistent excitation (3) is satisfied. The estimator described in (9) is employed to estimate the coordinates of each UGV0. The direct and indirect estimates are amalgamated to derive the ultimate fusion estimate $Z_{i}\left(k\right)$. According to Theorem 2, each UAV has its estimate $Z_{i}\left(k\right)$ that converges to the true coordinates $\psi_{i0}\left(k\right)$ of UGV0. Here, let $Z_{i}\left(k\right)$ represent the final fusion estimate. For simplicity, the corresponding real RL is $x_{i0}\left(k\right)$. Therefore, the evolution of estimation errors is represented by $∥err_{i0}\left(k\right)∥=∥Z_{i}\left(k\right)-x_{i0}\left(k\right)∥$. In the first and second simulations, the evolution of estimation errors for periodically and randomly switching simulations is depicted in Figure 9b,e and Figure 10a,b, respectively. It can be observed that even in the presence of noise, the fusion estimation error remains bounded uniformly. The RL fusion estimation errors are bounded with 0.6m in both cases. Our proposed method demonstrates robustness in simulation results, performing well in both prescribed trajectories and random scenarios.

In addition, in order to validate the effectiveness of the proposed RL fusion estimation scheme, we conducted comparative experiments. Figure 9a–c depict the simulation results of periodic switching, while Figure 9d–f show the results of random switching. Among them, (a), (d), and (c), (f) represent the corresponding error estimation curves under the methods in paper [30] and paper [31], respectively. (b) and (e) depict the error curves corresponding to the method proposed in this article. In the simulation results of the first simulation, a comparison of the graphs in (a) and (b) reveals that the error of the algorithm proposed in this paper is significantly smaller than the error of the method in paper [30]. Simultaneously, the algorithm proposed in this paper demonstrates faster convergence compared to that in paper [30]. Further, in the comparison between graphs (b) and (c), although the error in paper [31] is lower, its convergence speed is significantly slower.

A comparison of the results in Figure 9d,e, illustrating the first simulation’s outcomes, reveals that the algorithm proposed in this study exhibits a substantially lower error than the method presented in paper [30]. Additionally, our approach achieves faster convergence compared to paper [30]. While the error in [31] is lower, the rate of convergence is relatively slower, as evident in graphs (e) and (f). This demonstrates the robustness of our proposed cooperative RL method in various scenarios. Despite the errors in Figure 9a being one or two orders of magnitude larger than those in Figure 9b, they remain unacceptable as they are on the meter scale. The errors in (d) and (e) are also similar. In the presence of noise and data loss, the fusion estimator mitigates the impact of these factors to some extent. The additional information in indirect estimation contributes to expediting the convergence of RL fusion estimation. The RL fusion estimation scheme errors between Figure 9b,e in the first simulation and Figure 10a,b in the second simulation were compared. While the convergence speed of the second simulation is slower, it still satisfies the convergence criteria. It can be seen that based on the proposed method, RL fusion estimation scheme errors are bounded by 0.6 m. However, the errors using the method in [30,31] can be up to 1.5 m, which are much larger than those of the proposed method.

### 5.2. Integration of RL Fusion Estimation and Circumnavigation Control [25]

**Figure 11:**
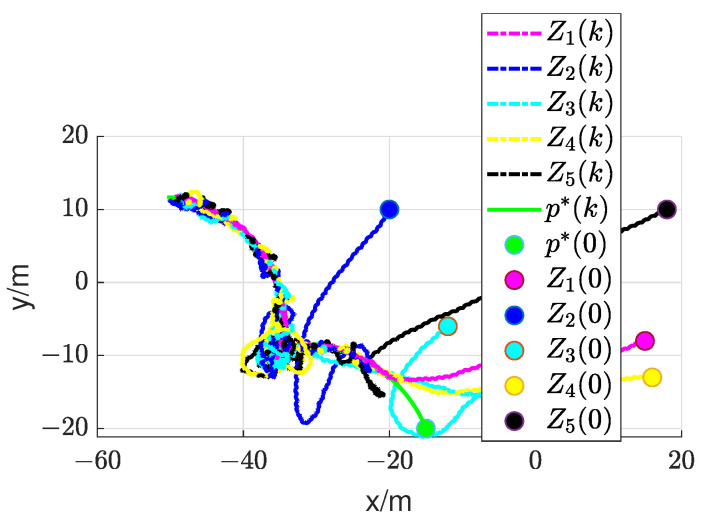
Simulation of a slowly drifting UGV0. The green circle denotes the initial position of UGV0, while the other circles indicate the initial positions of the UAVs.

**Figure 12:**
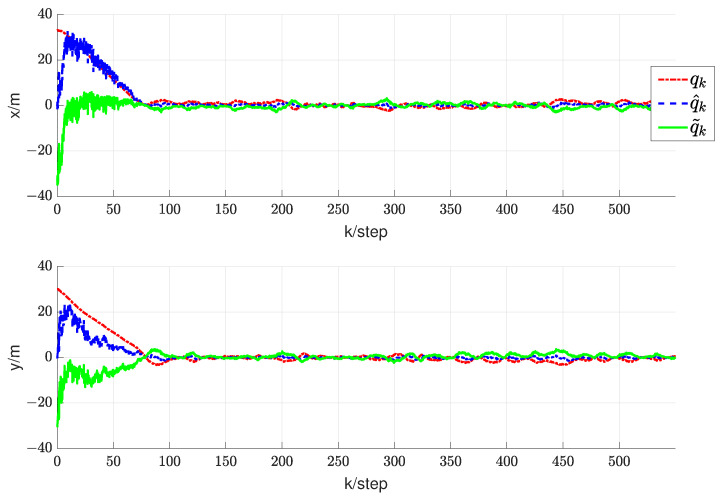
Trajectory diagram depicting the relative positions between UAV1 and UGV0.

**Figure 13:**
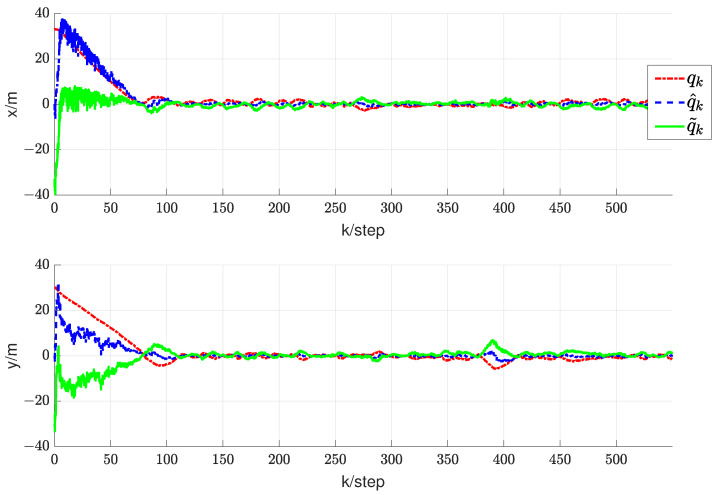
Trajectory diagram depicting the relative positions between UAV2 and UGV0.

**Figure 14:**
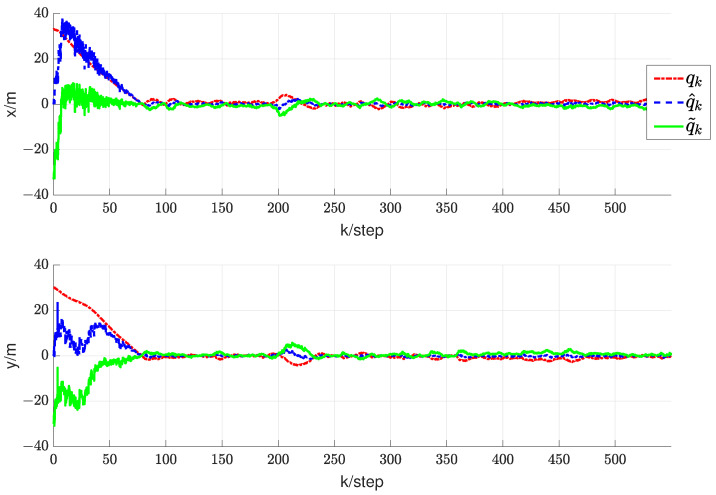
Trajectory diagram depicting the relative positions between UAV3 and UGV0.

**Figure 15:**
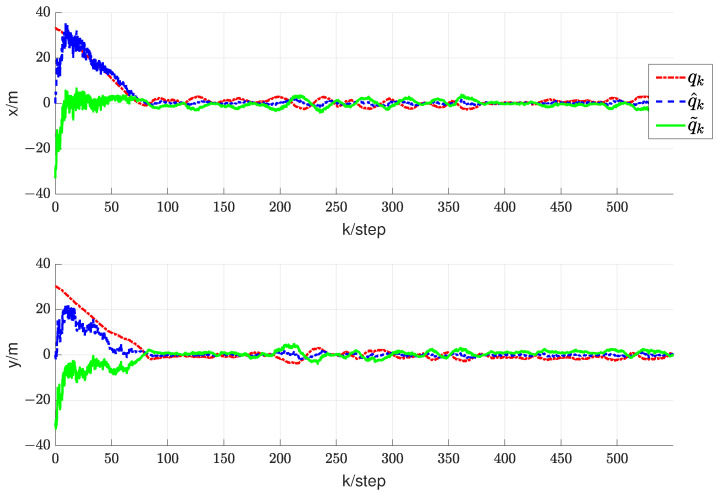
Trajectory diagram depicting the relative positions between UAV4 and UGV0.

**Figure 16:**
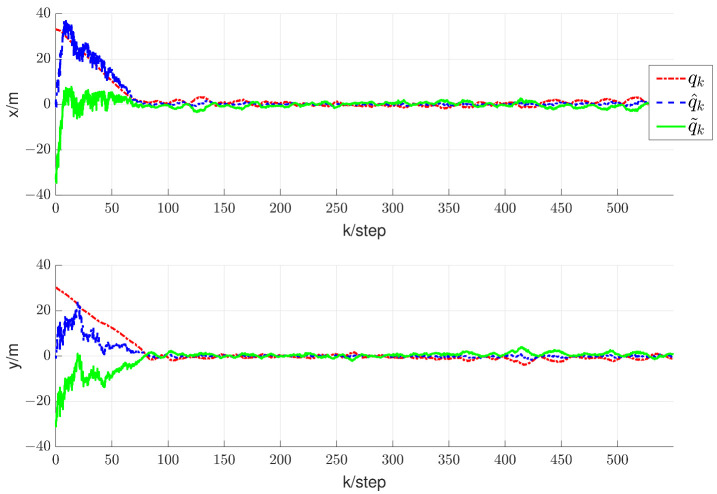
Trajectory diagram depicting the relative positions between UAV5 and UGV0.

In this section, we will validate the integrated RL and circumnavigation scheme proposed in Section 4 through numerical simulations to confirm its robustness. Consider a five-UAV team, where each UAV is able to measure the distance or angle to UGV0. The control goal is to enable five UAVs to hover around UGV0. In the third simulation, we verified the scheme proposed when UGV0 slowly drifts with an angular velocity equal to 0.005 rad/s. The UAV maintains its distance in a neighborhood of the desired range from UGV0. It is worth noting that the speed of UGV0 is consistently much lower than that of the other UAVs. In this scenario, we assume that $p^{*}\left(0\right)=$ [−20, −20] is the initial position of UGV0. The initial positions of the UAVs are set as [15, −8], [−20, 10], [−12, −6], [16, −13] and [18, 10], respectively. We can observe from Figure 11 that the UAV is capable of orbiting around UGV0 very well. Under these conditions, positive constants β=5 and α=4 are chosen to satisfy the given criteria. As depicted in Figure 12, Figure 13, Figure 14, Figure 15 and Figure 16, all trajectories converge to zero, validating our theoretical analysis.

**Figure 17:**
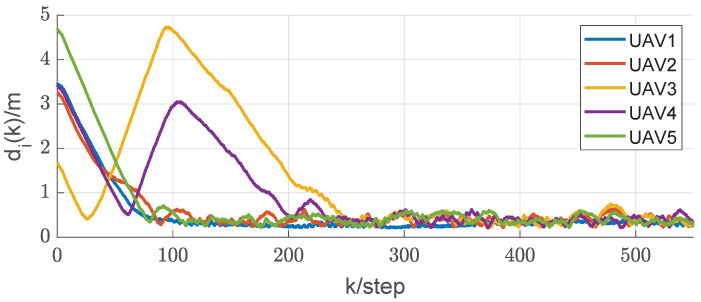
The rapid and stable approach of UGV0.

To demonstrate the effectiveness of the integrated RL and circumnavigation solution, tests were conducted with five UAVs. The graph in Figure 17 illustrates the reduction in distance between UAVs and UGV0. It is evident that the plane distance consistently decreases rapidly until the circumnavigation radius of 0.7 m is reached, greater than the minimum spacing of 0.2 m. In summary, the experimental results demonstrate the superior performance of the integrated RL and circumnavigation solution. It is anticipated that similar integration concepts can be further applied to more practical scenarios.

## 6. Conclusions

This paper proposes an RL fusion estimation and distance- or angle-based UAV circumnavigation control scheme that does not rely on infrastructure or the GPS. The proposed algorithm enables a UAV to locate UGV0 using only the distance or the azimuth angle, without any explicit distinction between the measured data. Integrated algorithms of RL estimators and circumnavigation controllers are explored. The concepts in this algorithm can also be extended to scenarios where the UAV’s motion is subject to non-holonomic constraints. A possible generalization is discussed in [32]. Finally, an integrated cooperative RL and circumnavigation control algorithm is proposed by combining the RL and circumnavigation control algorithms. Numerous simulation experiments have verified the effectiveness of the proposed algorithm.

Given the recent innovation and research outcomes in artificial intelligence (AI), we will explore opportunities to enable and improve functionalities in UAVs using AI techniques in the future. Both AI and UAV technology have become popular in recent times, along with research to bring the two fields together. Many problems inherent in UAVs today can be solved with the use of AI. Future research directions include addressing challenges in three-dimensional space and enhancing the algorithm’s convergence speed to reduce tracking and estimation errors within a limited timeframe. Additionally, considering a more general UAV model and accounting for the impact of noise are crucial aspects of future investigations. Another avenue for research involves exploring scenarios with UAV swarms and UGVs. Estimating the location of UGVs may be easier in the case of UAV swarms, as different UAVs can share their estimates of the UGV’s location. These shared estimates can expedite the estimation of UGV positions. Additionally, collision avoidance technology should be taken into account when deploying UAV swarms. Furthermore, it is essential to ensure that the distance between UAVs does not exceed their communication range, preventing the disruption of the connection link between them.

## Data Availability

Data are contained within the article.
